# Bleeding after Percutaneous Transhepatic Biliary Drainage: Incidence, Causes and Treatments

**DOI:** 10.3390/jcm7050094

**Published:** 2018-05-01

**Authors:** Keith B. Quencer, Anthony S. Tadros, Keyan B. Marashi, Ziga Cizman, Eric Reiner, Ryan O’Hara, Rahmi Oklu

**Affiliations:** 1 Division of Interventional Radiology, University of Utah Department of Radiology, Salt Lake City, UT 84108, USA; Keyan.Marashi@hsc.utah.edu (K.B.M.); Ziga.Cizman@hsc.utah.edu (Z.C.); Ryan.OHara@hsc.utah.edu (R.O.); 2 Department of Radiology, University of California-San Diego, San Diego, CA 92093, USA; anthonytadros@gmail.com; 3 University of Tennessee-West Cancer Center, Memphis, TN 38139, USA; ericreiner@snet.net; 4 Department of Vascular and Interventional Radiology, Minimally Invasive Therapeutics Laboratory, Mayo Clinic, Phoenix, AZ 85054, USA; Oklu.Rahmi@mayo.edu

**Keywords:** percutaneous transhepatic biliary drainage, iatrogenic hemobilia, pull-back cholangiogram, arterial-biliary fistula

## Abstract

Of all procedures in interventional radiology, percutaneous transhepatic biliary drainage (PTBD) is amongst the most technically challenging. Successful placement requires a high level of assorted skills. While this procedure can be life-saving, it can also lead to significant iatrogenic harm, often manifesting as bleeding. Readers of this article will come to understand the pathophysiology and anatomy underlying post-PTBD bleeding, its incidence, its varied clinical manifestations and its initial management. Additionally, a structured approach to its treatment emphasizing endovascular and percutaneous methods is given.

## 1. Introduction

Biliary drainage is needed in many situations, most often for cases of biliary obstruction or leak. While endoscopic and percutaneous drainage procedures have similar effectiveness and overall complication rates, endoscopic approach is the preferred initial option given greater patient comfort [1,2,3]. However, the common bile duct (CBD) cannot be cannulated endoscopically in approximately 8% of all attempted cases [4]. Additionally, when the anatomy of the upper gastrointestinal (GI) tract is surgically altered, such as after Roux-en-Y gastric bypass for obesity, Billroth II for gastric cancer or peptic ulcer disease and Roux-en-Y hepaticojejunostomy for liver transplantation, it is not possible to access the biliary system using traditional endoscopy techniques [5,6,7]. Percutaneous transhepatic biliary drainage (PTBD) is therefore the procedure of choice for these patients as well as for those in whom endoscopic cannulation of the CBD has failed and for those patients who have persistent obstruction or leak despite endoscopic therapy [8].

PTBD results in complications in up to 10% of cases, which range from minor (e.g., access site pain) to death [9]. Nonvascular complications of PTBD include pneumothorax, pleural effusion/empyema, biliary leak into the peritoneum and pancreatitis [9]. The reported rate of significant bleed after PTBD varies from 0.6% [10] to 12% [11]. Most large case series report a 2–2.5% significant bleeding rate [12,13]. The Society of Interventional Radiology (SIR) Quality Improvement Guidelines reflect this, with an expected 2.5% rate of bleeding complications and suggestion for departmental review when the bleeding rates exceed 5% [14]. Part of the differences reported in the literature of post-PTBD bleeding is the heterogeneous patient population and practice patterns. Another component is the varied definition of what constitutes significant bleeding after PTBD. The largest case series to date defined it as the need for transfusion of red blood cells and/or the need for transcatheter embolization [13] while another counted even transient small volume hemobilia as post-PTBD bleeding [15].

**Figure 1:**
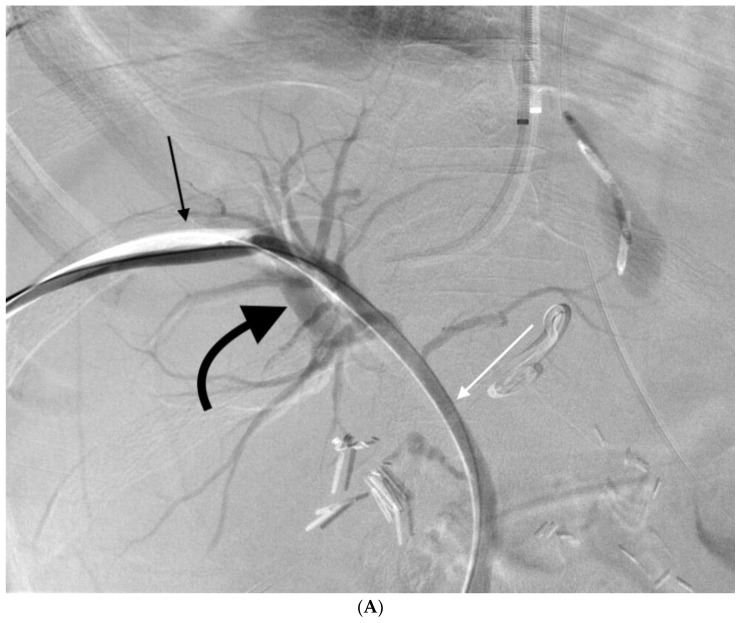

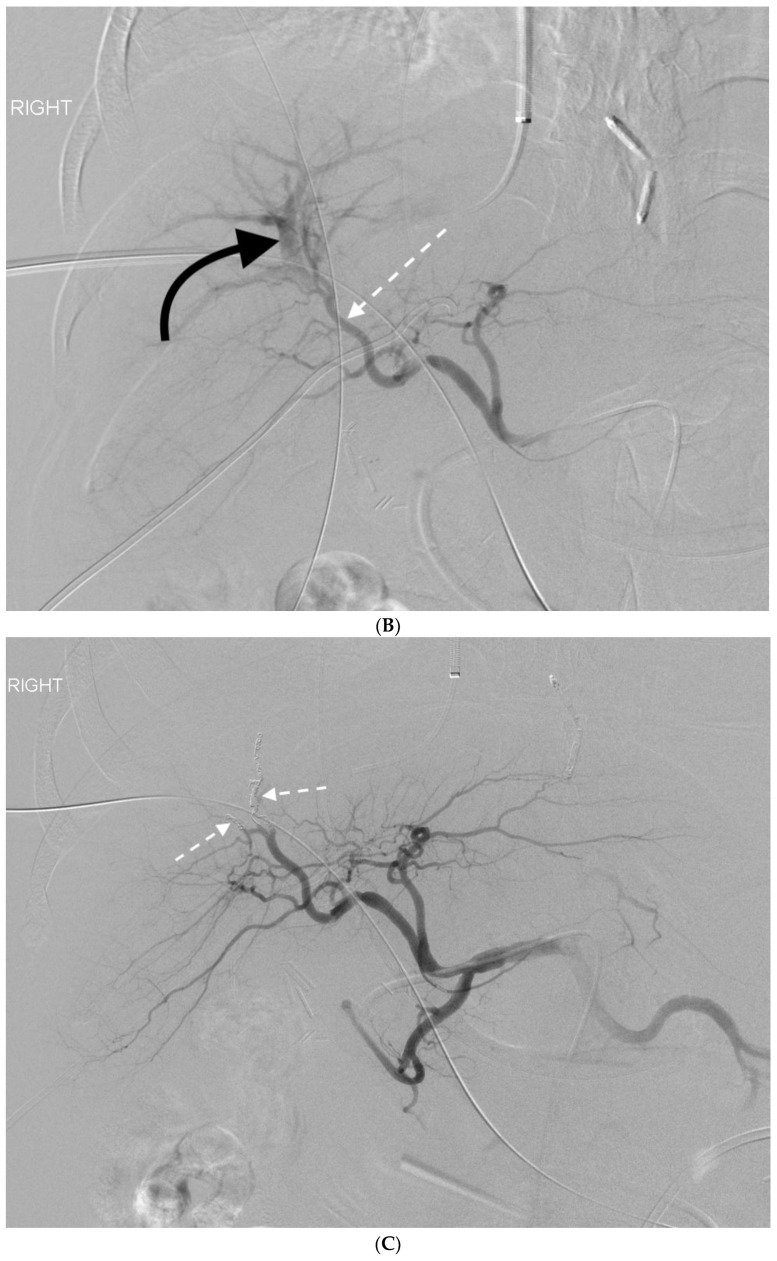
56-year-old male with combined arterial-portal and portal-biliary fistulas. Patient underwent percutaneous transhepatic biliary drain (PTBD) placement for a biliary leak. One week later, he developed significant hemobilia. (**A**) Pullback cholangiogram through a sheath (thin black arrow) showed opacification of the biliary tree (thin white arrow) and portal vein (curved black arrow). (**B**) Hepatic artery angiogram showed a fistula between a branch of the right hepatic artery (dashed white arrow) and branch of the right portal vein (curved black arrow). (**C**) After transarterial embolization with coils (dashed white arrows), the arterial-portal fistula resolved. A new biliary drain was placed with proximal side holes central to the site of portal vein traversal. The patient’s hemobilia resolved. The portal vein and hepatic artery travel alongside the bile ducts in the portal triad and are at risk of injury during PTBD placement.

The anatomy of the portal triad explains the risk of bleeding and elucidates the vessels at risk for injury during PTBD. The portal triad consists of the hepatic artery, portal vein (PV) and bile ducts. To puncture the biliary tree, the needle is necessarily in close proximity to both the hepatic artery as well as portal vein. Therefore, portal vein and hepatic artery injury make up the vast majority of vessels injured during PTBD (Figure 1). The hepatic vein is not part of the portal triad and is rarely injured during PTBD. When the hepatic vein is injured, it is usually as part of a significant injury that also involves the portal vein and/or hepatic artery [16,17]. 

Various endovascular and percutaneous interventions can be performed when post-PTBD bleeding occurs, all of which are highly effective in stopping the bleeding. If unsuccessful, surgical intervention such as vessel ligation or partial hepatic resection can be considered but these surgical procedures carry significant risks.

## 2. Pre-Procedure Work-Up and Risk Factors

The SIR categorizes new biliary tube placement as a procedure with significant bleeding risk and recommends the international normalized ratio (INR) to be <1.5 and the platelet count to be >50,000/mm^3^ [18]. Other authors suggest an INR <1.4 and platelets >70,000/mm^3^ for elective biliary drainage catheter placement with more lenient coagulation parameters (<1.6, >50,000/mm^3^ respectively) for emergent procedures [16]. When possible, aspirin and clopidogrel should be held for 5 days prior to the procedure; antiplatelet therapy with aspirin and/or clopidogrel has been found to more than double the risk for severe bleeding after PTBD [19]. However, waiting 5 days is often not feasible; platelet transfusion and/or administration of desmopressin are therefore warranted for urgent or emergent cases [20,21]. 

In addition to coagulopathy and antiplatelet use, there are other risk factors for increased risk of bleeding after PTBD.

- Current antiplatelet use
- Central biliary access
- Use of 18G (vs. 21/22G) access needle
- Left-sided drain placement *, * Inconsistently found in the literature
- Multiple passes
- Non-dilated system
- Cirrhosis
- Renal insufficiency
- Advanced age

These include left-sided puncture [22,23] and drainage of a non-dilated system [24]. The increased bleeding risk is explained by more central punctures in both of these groups. Left-sided drains typically access first- and second-order biliary radicles and relatively central punctures are often done out of necessity in non-dilated systems [24]. The finding of increased complications with left-sided drainage has not been borne out in more recent studies, however [25]. With central punctures, the adjacent hepatic artery and portal vein are larger and, when traversed, can result in increased risk of complications [25]. Of note, the rate of technical success of biliary drain placement decreases from approximately 95% to 65% when biliary ductal dilation is absent [14]. Another risk factor is the use of an 18-gauge needle instead of the more typical 21- or 22-gauge needle for accessing the duct. One study found a 7.7 times increased rate of significant bleeding when an 18-gauge needle was used [12]. The 18-gauge needle has been supplanted in modern practice by the 21- or 22-gauge Chiba needle [8]. Repeated needle passes through the liver capsule may lead to subcapsular hematoma. Other risk factors for post-PTBD bleeding include advanced age, cirrhosis, and chronic renal insufficiency [19]. 

## 3. Clinical Evaluation and Initial Treatment

**Figure 2:**
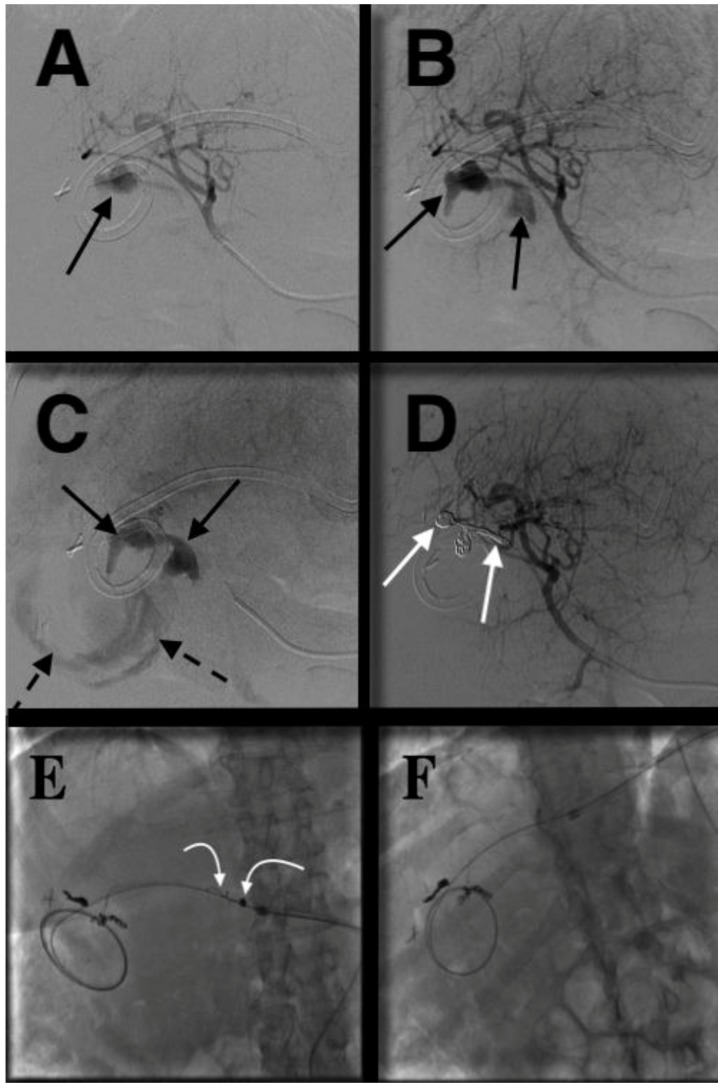
71-year-old female with PTBD erosion into hepatic artery and subsequent massive upper gastrointestinal bleed. Patient had a PTBD in place for 7 years following a common bile duct (CBD) injury sustained during cholecystectomy with subsequent hepaticojejunostomy and anastomotic narrowing. This stricture failed benign biliary stricture protocol dilation. She presented to the emergency department with hematemesis, hematochezia and hypotension. (**A**–**C**) Initial angiogram showed extravasation from the hepatic artery (black arrows) and into the small bowel (dashed black arrows). (**D**) After coils (white arrows) were placed distally and proximally, repeat hepatic artery angiogram showed cessation of extravasation. (**E**) During a routine biliary tube exchange 7 months later, some of the coils (curved white arrows) were noted to have migrated into the bile duct and along the biliary drainage catheter. (**F**) These coils were percutaneously removed using a snare.

Bleeding after PTBD presents in various ways [16] (Table 1). Patients may present with acute bleeding or with bleeding years after the initial placement (Figure 2) [26]. The severity of bleeding is widely varied and may be asymptomatic or fatal and may be intermittent or constant. Most commonly, bleeding is evident in the drainage bag. Hemobilia caused by PTBD characteristically presents as melena, but when it is brisk, such as in cases of significant hepatic artery injury, it may present as hematochezia [27]. Bleeding may occur at the skin site, within the chest wall/pleural space manifesting as hemothorax or may be subcapsular, manifesting as hemoperitoneum. Finally, the bleeding may be pulsatile or a slow ooze. It may be bright red or dark. These varied clinical findings are all clues as to the site and vessel of injury and drive the work-up and treatment of post-PTBD bleeding. Signs of hepatic arterial bleeding include pulsatile bleeding, bright red blood in the drainage bag, hemodynamic instability and significant drop in hematocrit level. Portal vein bleeding manifests with dark blood in the drainage bag without hemodynamic instability. Portal vein bleeding is frequently intermittent, which occurs when the most peripheral side hole slides in and out of the portal vein vessel with changes in position. Bleeding from a hepatic artery source is usually constant. Clotted blood in the biliary tree may also worsen obstructive jaundice, and if present in the CBD, can lead to pancreatitis [28]. 

Hemodynamic instability after PTBD is not always related to bleeding. In fact, sepsis occurs in approximately 5% of cases. Additionally, sedation and general anesthesia can also have lingering effects reducing blood pressure. When there is significant post-PTBD bleeding, coagulation parameters, complete blood count (CBC) and type and crossmatch should be immediately obtained. Coagulopathy should be promptly corrected. Two large bore IVs should be placed for anticipated transfusion. Vital sign assessment, pulmonary and abdominal physical examination and direct visualization of tube entry into the skin are all essential parts of a focused physical exam. 

Bleeding may be as benign as venous skin oozing at the tube entry site, which can be treated by manual pressure, a pressure dressing, application of a hemostatic pad and/or placement of a purse-string suture around the tube. Once it is determined that the site of bleeding is not from a superficial skin vein, the differential diagnosis includes an intercostal artery, hepatic artery or portal vein bleed. As mentioned previously, hepatic vein injury is rarely the sole cause of post-PTBD bleeding, but can be seen as part of a complex injury that involves the hepatic artery and/or portal vein.

The intercostal artery runs inferior to each respective rib, and risk of injury to the intercostal artery can be reduced by crossing above rather than below the rib [30]. The intercostal artery, however, can be redundant/tortuous within the intercostal space, especially in older patients, and is at risk for injury even when puncture is performed immediately above the rib [31]. Clinically, intercostal injury should be suspected with hemodynamic instability and/or falling hematocrit without significant blood within the drainage bag or hemobilia. Injury to this artery will manifest as an expanding chest wall hematoma or hemothorax. When intercostal artery bleeding is suspected, computed tomography angiography (CTA) can be helpful to confirm the diagnosis and elucidate the exact location of the bleed. CTA should only be done if the patient is stable enough to travel to the CT scanner before presenting to the interventional radiology suite for angiogram and embolization. Endovascular embolization has a high success rate in stopping bleeding from an intercostal artery [32]. Chest tube placement and/or thoracostomy may be needed in cases of large hemothorax to prevent the subsequent development of a fibrothorax [33]. 

## 4. Management of Portal Vein and Hepatic Artery Injuries

### 4.1. Portal Vein

**Figure 3:**
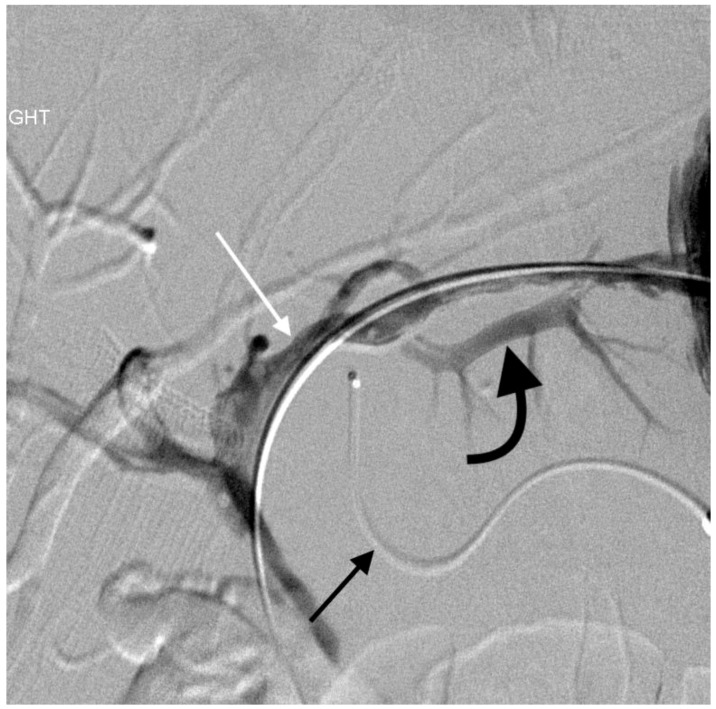
56-year-old female with portal vein injury from left-sided PTBD placement. Patient underwent emergent PTBD placement for acute cholangitis. After placement, she had dark blood coming from the biliary drain, with a 20% drop in her hematocrit from baseline. She had hemodynamic instability. It was unclear based on this clinical picture whether the patient had an arterial or venous source of bleeding. Hepatic artery angiogram was followed by pullback cholangiogram. The former showed no signs of arterial injury, even after the biliary drain was removed over a wire. Pullback cholangiogram showed traversal of a left-sided portal vein branch (curved black arrow). White arrow—left hepatic duct; straight black arrow—microcatheter for concurrently performed hepatic artery angiogram. The bleeding was successfully treated with drain upsizing and placement of most proximal side holes more centrally. Common bile duct stones were removed endoscopically, and the drain was removed 7 weeks later. At that time, repeat pullback cholangiogram showed no opacification of the portal vein secondary to epithelialization of the tract.

Portal vein injury from PTBD is caused by access needle traversing the portal vein prior to entering the adjacent bile duct (Figure 3). It should be suspected when bleeding occurs with dark blood and without frank hemodynamic instability or a large drop in hematocrit levels. Hemodynamic instability can occasionally be seen when a large portal vein branch is traversed or in the setting of portal hypertension. At the bedside, the tube should be capped to provide partial tamponade by stopping the low-pressure egress into the drainage bag. This slows ongoing blood loss while arranging for patient transport to the interventional radiology suite. In general, CTA is not sensitive in identifying either portal venous or hepatic artery biliary bleeding [34]. Once in interventional radiology, a pullback cholangiogram is done to confirm the suspected diagnosis and assess the size/location of the portal vein branch that has been traversed. Pullback cholangiogram is performed by doing the following. First, the tube is removed over a wire. Then, a sheath, which is 2 French smaller in diameter than the existing tube is placed over the wire. Finally, the sheath is then slowly and steadily pulled back over the wire while injecting contrast while acquiring digital subtraction images. If a large/central portal vein is opacified, tamponade by gentle balloon inflation at 3–5 atmospheres across the site of PV injury can be done until more definitive therapy is sought. Alternatively, tamponade of the bleed can be achieved with re-advancement of the sheath across the site of injury or by placement of a drain with side holes central to the site of injury. 

Central PV injury needs to be definitively managed; various treatment strategies are available and the best choice depends on the site of injury and patient condition. If portal vein access can be obtained, options include placing a stent graft across the site of injury [35] or coiling the effected portal vein [36]. Coiling within a central portal vein risks infarction of the subtended hepatic parenchyma. Other options include placing coils via the percutaneous drain access so that they straddle the site where the portal vein is traversed [37] and stent graft placement within the biliary system across the site of portal-biliary fistula [38]. If, as is more typically seen, the traversed vessel is a small peripheral PV branch, this is treated by tube upsizing, ensuring the side holes are central to PV branch. This only temporizes the problem but also fixes the underlying abnormality by allowing epithelialization of the hepatic parenchymal tract and/or thrombosis of the portal vein branch. Alternatively, if new PTBD access is gained, coils can be placed across the entire transhepatic tract. 

### 4.2. Hepatic Artery

Clinically, hepatic artery bleeding after PTBD presents with bright red, sometimes pulsatile blood through the tube, often accompanied by hemodynamic instability. Significant fall in hematocrit level (>13% from baseline) is a highly specific but relatively insensitive sign of hepatic artery injury [12]. Delayed hepatic artery bleeding can occur secondary to PTBD stent erosion into the hepatic artery (see Figure 2). If arterial bleeding is suspected, drain injection and/or pullback cholangiogram should be avoided as the injected contrast will obscure the subsequent angiogram as well as delay definitive treatment, which is trans-arterial embolization. The clinical picture, however, is often not clear and hepatic artery injury may be mistaken for portal vein injury. In these cases, pullback cholangiogram may be initially performed. Pullback cholangiogram will rarely show direct hepatic artery injury, manifested by opacification of the artery peripheral to the site of injury. This is because of the small size of the vessel as well as the fact that, to avoid precipitating sepsis, one should not inject the sheath with the requisite pressure required to overcome systemic arterial pressure. There is, however, an indirect sign of hepatic artery injury on pullback cholangiogram wherein only the peripheral bile ducts are opacified. This is a result of pressurization and hepatofugal flow within the biliary tree related to arterial-biliary communication and has been termed the “absent central bile duct sign” [16]. 

If, after performing initial hepatic artery arteriography in different obliquities, no findings may be seen to explain the suspected hepatic artery injury, pulling the PTBD out over a wire will unmask any tamponade effect provided by the drain. Sandover et al. demonstrated that this maneuver unmasked an arterial bleed in 42% of initially “negative” arteriograms [29]. 

**Figure 4:**
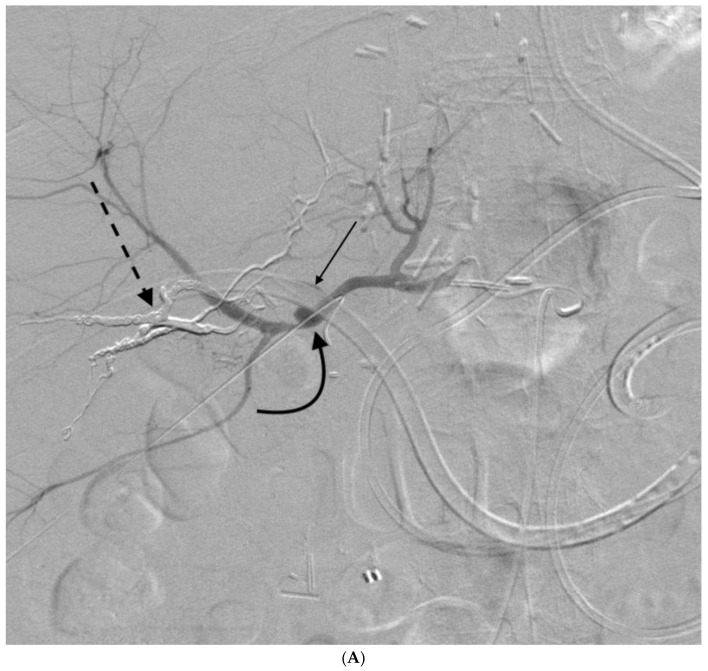

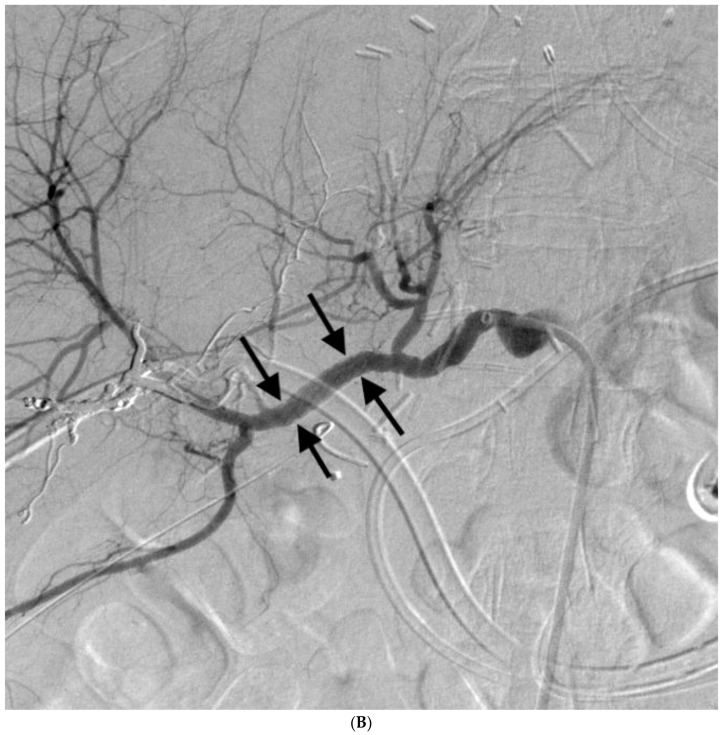
61-year-old female with hepatic artery pseudoaneurysm caused by PTBD, treated with stent graft placement. Patient underwent deceased donor liver transplant for hepatitis C cirrhosis. She subsequently developed large volume bleeding from her biliary drainage catheter. (**A**) Angiogram showed a hepatic artery to portal vein fistula that was treated with coil and glue embolization (dashed black arrow) as well as a hepatic artery pseudoaneurysm (curved black arrow) adjacent to the crossing biliary drainage catheter with extravasation seen around the catheter (thin black arrow). (**B**) Given the patient’s favorable anatomy, a covered stent (straight black arrows) was placed with desired exclusion of the pseudoaneurysm and cessation of hemobilia.

When hepatic artery injury is identified, treatment options include stent graft placement and embolization. Stent graft placement across the site of injury is feasible only with central injury and favorable anatomy [39,40]. Stent graft should extend 5–10 mm proximal and distal to the site of injury (Figure 4). 

**Figure 5:**
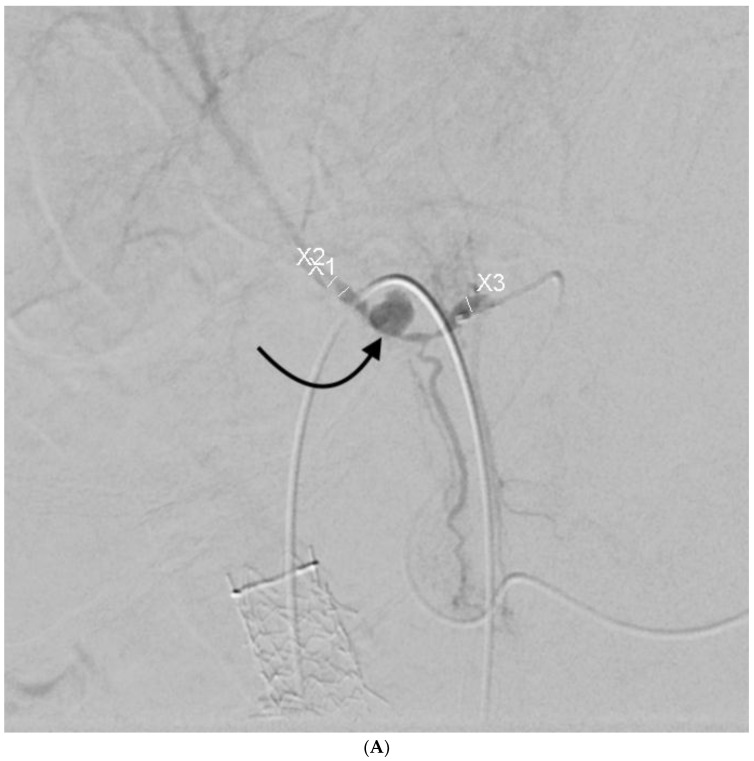

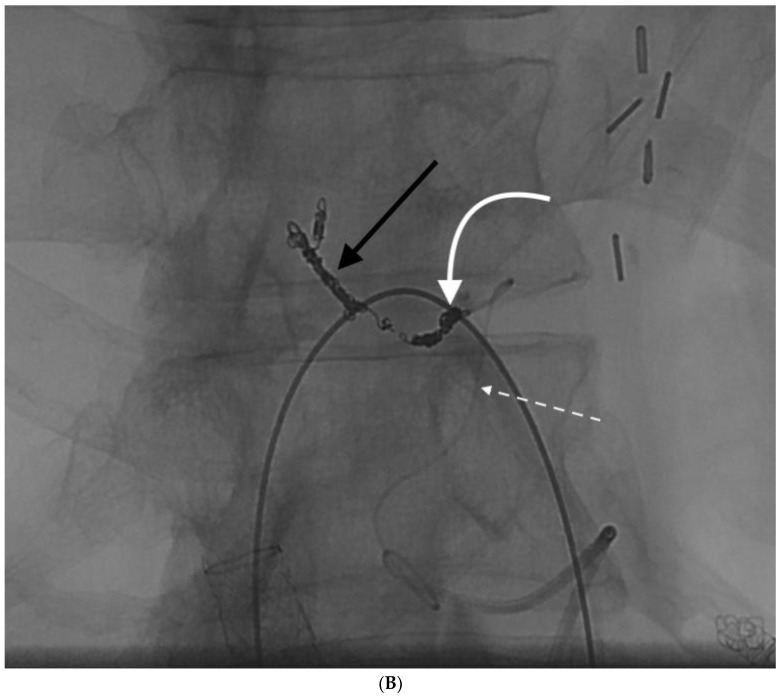
58-year-old male with a pseudoaneurysm caused by PTBD that was treated with coil embolization. This patient had a history of ampullary carcinoma for which he underwent Whipple procedure complicated by bile leak at the choledochojejunostomy site. PTBD was placed with multiple subsequent episodes of hemobilia, which were initially treated with tube upsizing. (**A**) Angiogram 3 weeks after initial drain placement with drain removed over a wire showed a large pseudoaneurysm (curved black arrow) arising from the left hepatic artery. (**B**) Coils were placed distal (straight black arrow) and proximal (curved white arrow) to the pseudoaneurysm using a microcatheter (dashed white arrow). The hemobilia was successfully stopped.

Embolization is more commonly performed, most often with coils (see Figure 2 and Figure 5). Coils are preferred over liquid embolics as the latter may lead to excessively distal embolization with risk of abscess formation [29,41]. Additionally, liquid embolics may extend into the biliary tree when arterial-biliary communication is present. Ideally, coil embolization is started just beyond the site of arterial injury, across and proximal to the site of injury [42]. Embolization only proximal to the site of injury risks continued bleeding as intrahepatic collateral circulation could lead to continued perfusion to the site of injury [43,44]. Embolization should be done as selectively as possible; biliary obstruction, even if relieved, can compress the adjacent portal vein [29,45]. This makes the hepatic parenchyma predominantly dependent on hepatic arterial supply. Therefore, ischemia of the tissue supplied by the hepatic artery can ensue after arterial embolization. Given that the biliary tracts of patients with PTBD drains are chronically colonized, infarction predisposes to subsequent abscess formation [46]. Finally, one must be conscious that the biliary tree of a hepatic transplant is highly susceptible to necrosis if there is a reduction in hepatic artery supply [47]. Hepatic artery embolization in these patients may lead to biliary necrosis. Therefore, hepatic artery embolization in transplant patients should be done as selectively as possible. 

**Figure 6:**
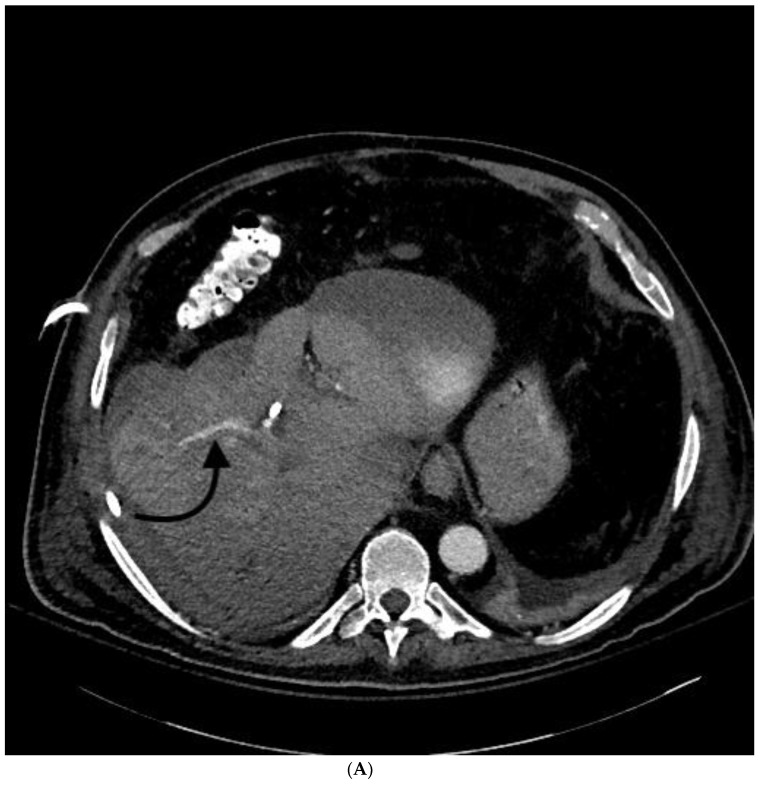

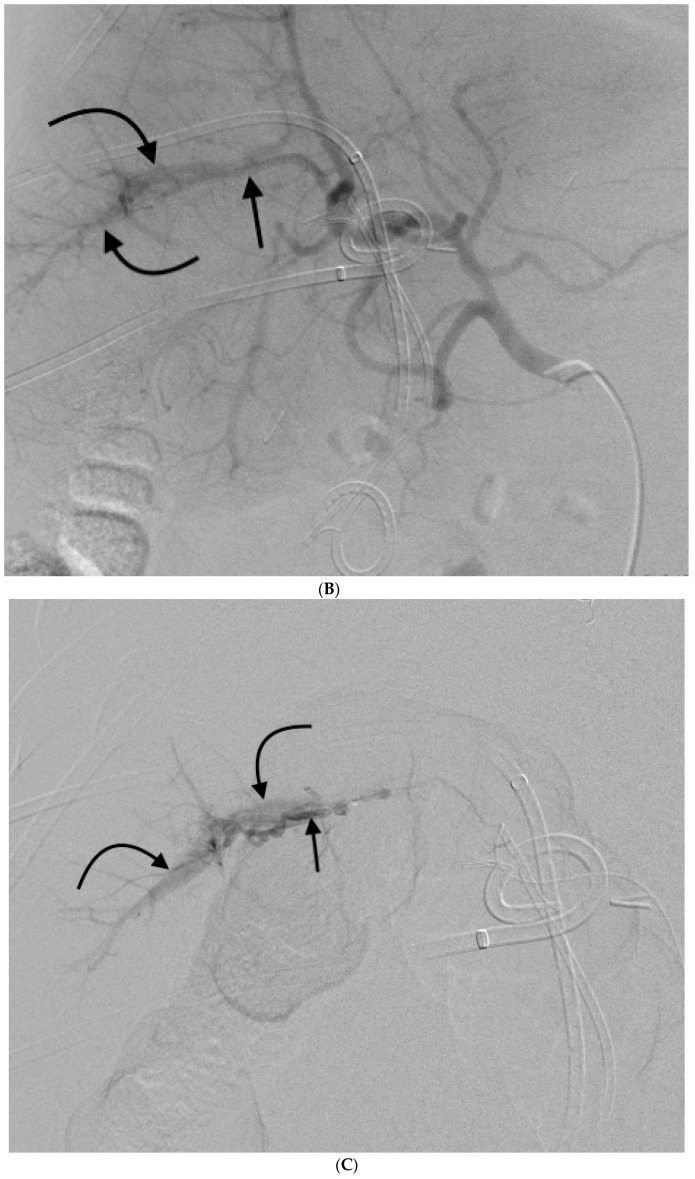

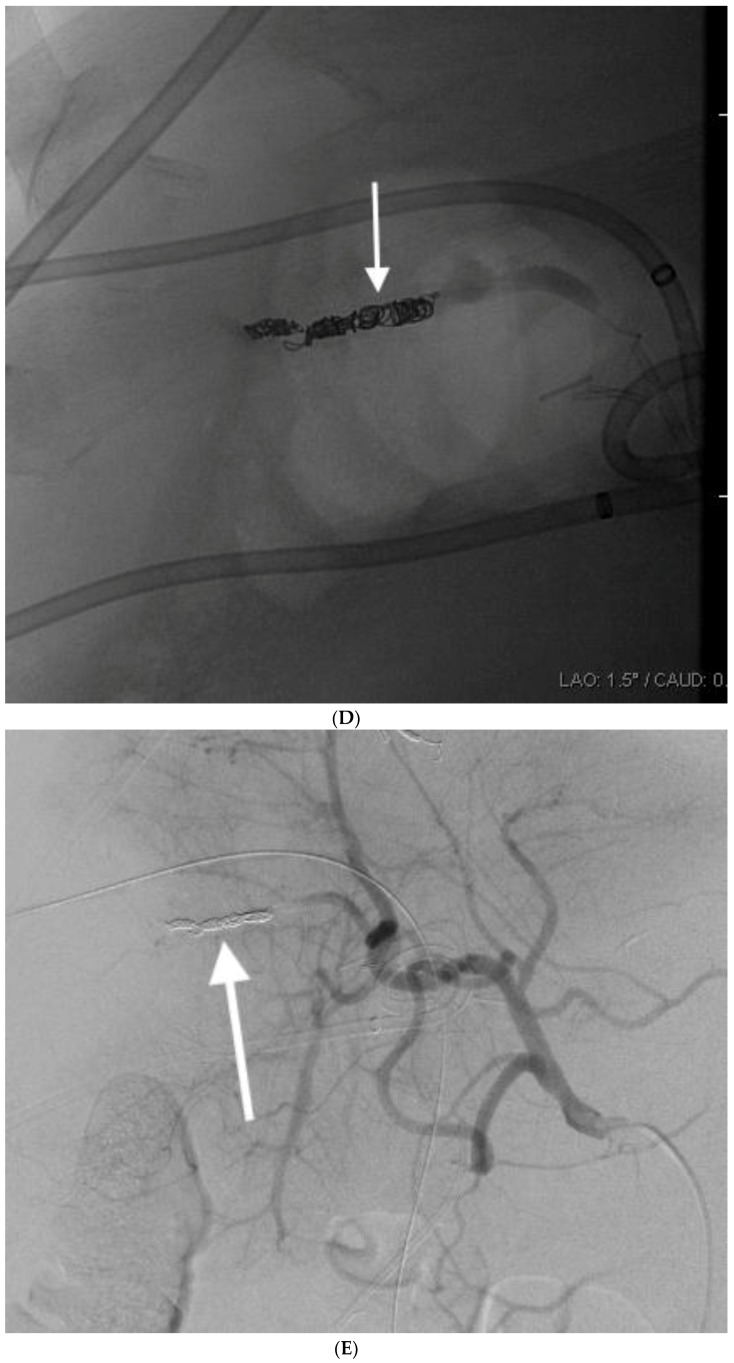
72-year-old male with a hepatic artery to portal vein fistula. This patient underwent PTBD placement for biliary diversion after a cholecystectomy led to a cystic duct stump leak. He had intermittent episodes of small volume hemobilia. (**A**) Computed tomography angiography (CTA) was performed and showed early opacification of a portal vein branch (curved arrow) for which he was referred for hepatic arteriogram. Celiac (**B**) and selective right hepatic (**C**) artery angiograms demonstrated hepatic artery (straight black arrows) to portal vein (curved black arrows) fistula, relatively remote from the PTBD. This injury was therefore thought to be related to initial needle passes. (**D**) The hepatic artery supplying this fistula was coil-embolized (white arrows). (**E**) Repeat hepatic angiogram demonstrated cessation of the arteriovenous fistula.

Hepatic artery to portal vein fistula can be caused by PTBD and may manifest with hemobilia [17,48] (Figure 6). While angiographic evidence of contrast entering the biliary system may not be seen, arterial-portal fistulas are a sign of penetrating vessel injury involving both the hepatic artery and portal vein. While peripheral and incidentally noted arterial-portal fistulas may be managed conservatively, those which cause bleeding or are central should be intervened upon [49]. Central arterial-portal fistulas may eventually result in portal hypertension [50]. Angiographic and clinical success is reported to be 90–95% after transcatheter embolization [17].

## 5. Conclusions 

**Figure 7:**
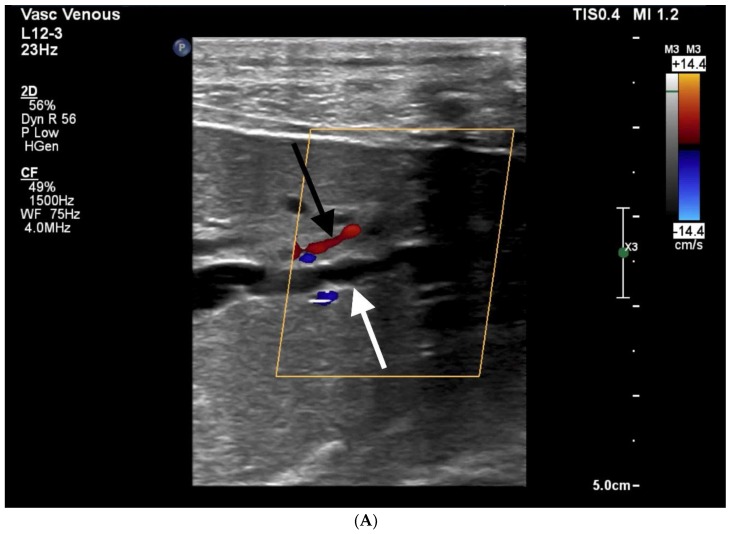

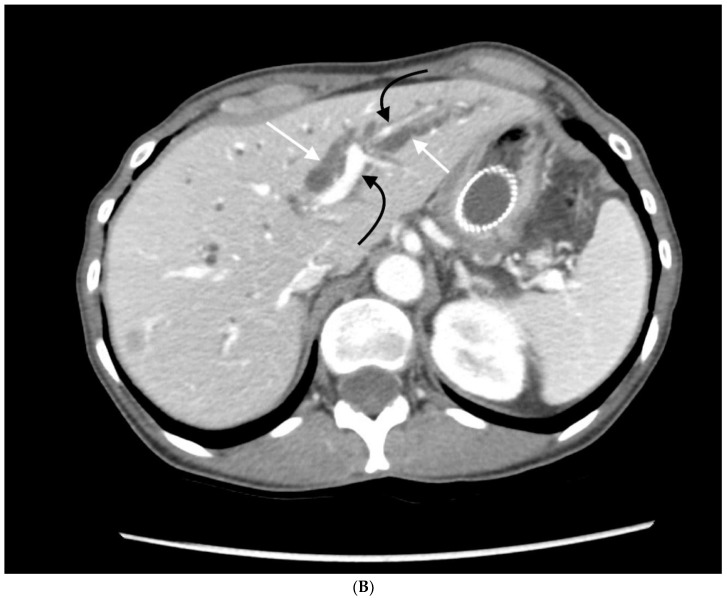

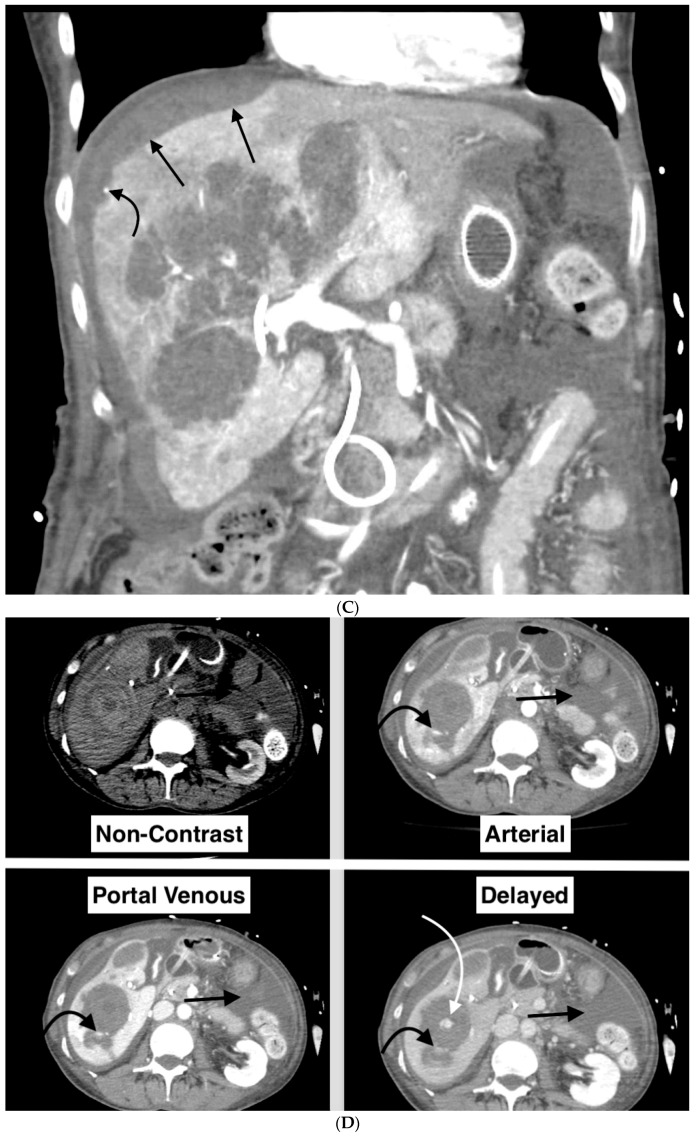
69-year-old female with PTBD complicated by liver lacerations and subsequent death. Patient had malignant obstructive jaundice secondary to esophageal cancer. Pre-procedure images, including ultrasound (**A**) and CT (**B**), showed significant biliary ductal dilation (white arrows). Note the expected proximity of the hepatic artery (straight black arrow in **A**) and portal vein (curved black arrows in **B**). Despite seemingly uncomplicated placement of bilateral biliary drainage catheters, the patient decompensated 3 h later. (**C**,**D**) Multiphase CT showed hemoperitoneum (straight black arrows), focal areas of arterial extravasation (curved black arrows) and venous pooling (curved white arrow in **D**). Multiple large intrahepatic lacerations were seen. Given patient’s overall prognosis and multifocal liver injuries, the patient was transitioned to comfort care and passed away 48 h later.

PTBD is a commonly performed interventional radiology procedure and requires significant intra-procedural skill. Clinical acumen and familiarity with the gamut of potential complications are necessary to provide effective post-procedure care. One complication, which occurs in approximately 2–3% of all PTBD placements, is significant bleeding and can result in patient death (Figure 7). Clinical signs and symptoms provide clues as to what vessel was injured, which dictate the next steps for further imaging and treatment. Pullback cholangiogram should be done when portal vein injury is suspected as this will confirm the diagnosis and show the site of vessel traversal, thereby guiding further interventions. When hepatic arterial injury is suspected, arteriography should be performed. Selective trans-arterial embolization is a highly effective and safe treatment for post-PTBD bleeding from hepatic artery injury. 

## Figures and Tables

**Table 1:** Clinical signs of portal vein versus hepatic artery injury, noting that significant overlap may be seen in the clinical picture. * A drop in baseline hematocrit by 13% or more was 94% specific for hepatic artery injury but only 10% sensitive [12]. If portal vein injury is suspected, the best imaging modality to confirm this is a pullback cholangiogram. If hepatic artery injury is suspected, angiogram should be performed without delay. If initial angiogram is negative, the biliary drain should be removed over a wire, a maneuver which has been shown to increase sensitivity of the angiogram 1.7 times fold [29].

Portal Vein Injury	Hepatic Artery Injury
Intermittent bleeding	Constant bleeding
Dark blood	Pulsatile bleeding
Typically hemodynamically stable	Bright red blood
No large drop in hematocrit	Falling hematocrit by >13% of baseline *
	Hemodynamic instability
Melena	
